# Curcumin suppresses EMT to alleviate oral submucous fibrosis progression through XIST/miR-25-3p-mediated inactivation of the TGF-β1/Smads signalling pathway

**DOI:** 10.1016/j.identj.2025.103975

**Published:** 2025-10-31

**Authors:** Qian Min, Yao Liu, Jian Liu, Yue Zhao, Jian Qin

**Affiliations:** a Department of Endodontics and Dentistry, Changsha Stomatological Hospital, Affiliated Stomatology Hospital of Hunan University of Chinese Medicine, Changsha, Hunan, China; b Bafang Outpatient Department, Changsha Stomatological Hospital, Affiliated Stomatology Hospital of Hunan University of Chinese Medicine, Changsha, Hunan, China; c Department of Periodontics, Changsha Stomatological Hospital, Affiliated Stomatology Hospital of Hunan University of Chinese Medicine, Changsha, Hunan, China

**Keywords:** Oral submucous fibrosis, curcumin, XIST, miR-25-3p, EMT, TGF-β1 /Smads

## Abstract

**Background:**

Oral submucous fibrosis (OSF) is a chronic oral disease with a tendency to carcinogenesis. At present, the treatment of OSF is limited and ineffective, and there is an urgent need to find new effective drugs and mechanisms of action.

**Methods:**

Arecoline was used to induce human oral mucosal fibroblasts (HOFs) to establish OSF cell model. The effects of curcumin on the proliferation, migration and invasion of HOFs were detected by CCK-8 and Transwell. Western blot (WB) was used to detect the effect of curcumin on epithelial-mesenchymal transition (EMT) process and TGF-β1/Smads signalling pathway. The regulation of XIST and miR-25-3p expression by curcumin was detected by qRT-PCR, and the role of XIST/miR-25-3p axis in curcumin function was investigated by rescue assay to detect cell viability, EMT progression and TGF-β1/Smads signalling pathway.

**Results:**

Curcumin significantly inhibited HOF cell viability (about 58%) and EMT process, down-regulate the expression of mesenchymal markers N-cadherin and Vimentin (over 40%), up-regulate the expression of epithelial marker E-cadherin (twice), and inhibit the activation of TGF-β1/Smads signalling pathway (about 50%). Curcumin regulated the expression levels of XIST (downregulated by approximately 58%) and miR-25-3p (increased by more than twice), and XIST regulated the expression of miR-25-3p through sponge adsorption. Further studies confirmed that curcumin regulated HOF cell activity, EMT process and TGF-β1/Smads signalling pathway through the XIST/miR-25-3p axis.

**Conclusions:**

Curcumin effectively inhibits EMT by inactivating the TGF-β1/Smads pathway through regulating XIST/miR-25-3p, and thereby alleviating the progression ofOSF. The study results further reveal the mechanisms underlying the function of curcumin, and may provide novel targets and ideas for OSF therapy.

## Introduction

Oral submucous fibrosis (OSF) is a chronic and progressive disease that seriously endangers oral health. Its pathological features are excessive hyperplasia and degeneration of fibrous tissue in the lamina propria and submucosa of the oral mucosa, leading to loss of oral mucosa elasticity and limitation of mouth opening, which seriously affects the chewing, swallowing and language functions of patients.^1^^,^^2^ OSF is widely recognised as condition precancerous to oral cancer. The malignant transformation rate of OSF ranges from 1.2 to 23% worldwide.^3^ Epidemiological studies have shown that the incidence of OSF is significantly increased in Southeast Asia, South Asia, Hunan, Hainan, and other areas with high consumption of betel nut. Long-term chewing of betel nut is recognised as the main pathogenic factor of OSF.^4^ Arecoline can induce abnormal proliferation and collagen synthesis of oral mucosal fibroblasts, promote inflammatory response and oxidative stress, and then accelerate the process of the disease.^1^^,^^5^ The core pathogenesis of OSF involves continuous inflammatory stimulation, oxidative stress and abnormal fibrosis repair process,^6^ among which the excessive activation of epithelial-mesenchymal transition (EMT) and transforming growth factor-β1 (TGF-β1) signalling pathway is the key link driving fibrosis progression.^7^ At present, the clinical treatment methods for OSF include surgical resection, drug intervention and physical therapy, but these methods have limitations, such as large surgical trauma, easy recurrence, and uncertain drug efficacy. Therefore, it is urgent to explore the pathogenesis of OSF and find safe and effective therapeutic drugs and intervention targets.

Curcumin is a natural polyphenolic compound extracted from Curcuma longa, which has attracted much attention in the field of disease treatment due to its wide range of biological activities. Studies have shown that curcumin has significant anti-inflammatory, anti-oxidation and anti-fibrosis effects.^8^^,^^9^ It has been reported that curcumin can inhibit the activation of TGF-β1/Smads pathway^10^^,^^11^ and block the EMT process, showing good application prospects in a variety of fibrotic diseases.^12^^,^^13^ However, the specific mechanism of curcumin in OSF remains unclear, which is a key knowledge gap.

Long non-coding RNA (lncRNA) is a class of RNA molecules with a length of more than 200 nucleotides and no protein coding function. lncRNA can act as a competing endogenous RNA (ceRNA) to adsorb miRNA, indirectly regulate the expression of miRNA target genes, and then affect cell function and disease progression.^14^ In OSF, lncRNA XIST is upregulated and participates in the activation of myofibroblasts, and its interaction with miR-25-3p has been reported in other diseases.^15^^,^^16^ The abnormal activation of fibroblasts is the core pathological feature of OSF. XIST, as a validated lncRNA related to fibrosis, is abnormally upregulated in OSF, providing a direct pathological basis for its involvement in disease regulation. The excessive activation of the TGF-β1/Smads pathway is a key driving mechanism for the initiation of the EMT process and the excessive deposition of extracellular matrix in OSF. It is worth noting that miR-25-3p interacts with the core mediator of fibrosis, TGF-β1,^17^, ^18^, ^19^ but the role of the XIST/miR-25-3p axis in OSF especially its relationship with the TGF-β1/EMT signalling pathway has not yet been studied.

Based on the above background, the present study aimed to reveal whether curcumin could alleviate OSF fibrosis progression by regulating the TGF-β1/Smads pathway mediated by XIST/miR-25-3p to inhibit EMT. The effect of curcumin on XIST/miR-25-3p/TGF-β1/Smads/EMT regulatory axis was explored by cell experiments. This study may provide a new theoretical basis and potential therapeutic targets for the treatment of OSF, and lays a foundation for the development of curcumin-based treatment strategies for OSF.

## Materials and methods

### Cell culture and arecoline treatments

The Human Oral Mucosa Fibroblasts Cells (HOFs) (CP-H205, Wuhan Punosai Life Technology CO., LTD.) were seeded in culture flasks and cultured in DMEM medium (Hyclone) supplemented with 10% foetal bovine serum (Gibco) in a cell incubator at 37°C with 5% CO₂. Cells were grown to 80% digested and passaged. In this study, all the HOFs used were subjected to experiments with cells from the 3rd to 6th generations. This passage range is within the stable period of cell biological characteristics, which can avoid cell phenotypic drift caused by excessive passage times. Before using the cells, they were all tested for mycoplasma: using the PCR method (item number: CA1081, Solarbio) for detection, ensuring that the experimental cells were free from mycoplasma contamination. At the same time, we identified the identity of the cells: through short tandem repeat sequence (STR) typing detection (completed by Wuhan Promesa Life Science Technology Co., Ltd.), the results were consistent with the standard STR pattern of this cell line (matching rate > 95%), confirming the accuracy of the cell source and identity.

Arecoline (No. 63-57-2, Chengdu Durst Biotechnology Co., LTD.) was prepared into 1 mg/mL mother liquor with sterile water, filtered to remove bacteria (0.22 µm), and stored at -20°C in the dark. When the cell density of HOFs was about 90%, the OSF model was induced by adding DMEM medium containing arecoline (40 µg/mL) for 24 hours.^20^

### Cell treatments

Curcumin (458-37-7, Chengdu Durst Biotechnology Co., LTD.) was dissolved in DMSO, vortexed until completely dissolved, and prepared into 10 mmol/L mother liquor. The liquid was then filtered with 0.22 µm microporous filter membrane and stored at -20°C in the dark. Curcumin mother liquor was added to DMEM high glucose complete medium containing 40 µg/mL arecoline and mixed to prepare a working solution with a concentration of 40 µmol/L.^21^^,^^22^

In order to regulate the expression of miR-25-3p and XIST, miR-25-3p mimic, miR-25-3p inhibitor, mimic NC, inhibitor NC, and pcDNA3.1-XIST were synthesised by GenePharma. Lipofectamine 3000 reagent (Invitrogen Life Technologies Inc.) was combined with the synthesised vector. The mixture was mixed for 20 min at room temperature, and then the mixture was added to HOFs cultured in serum-free medium. After 6 hours, the culture was replaced with complete medium containing 10% foetal bovine serum and continued for another 18 hours.

### Cell viability

Cells in the logarithmic growth phase were selected and seeded in 96-well plates. After 24 hours of incubation, cell counting kit (CCK)-8 buffer was mixed with DMEM at a ratio of 1∶9 and added to 96-well plates. The cells were incubated at 37°C for 2 hours, and the OD value at 450 nm was determined using a microplate reader.

### Transwell assay

Transwell chambers (Costar, Corning Inc) were used to detect cell migration using an 8-um pore size membrane. Matrigel (BD Biosciences) was precoated on Transwell chambers to detect cell invasion. The upper chamber was added with 2 × 10^5^ HOFs in 200 µL of serum-free medium, and the lower chamber was filled with 600 µL of complete medium. After 24 hours of incubation, non-invasive cells on the upper surface of the filter were wiped with a cotton swab. Meanwhile, cells that had passed through the membrane were fixed with methanol, stained with crystal violet, and photographed with a digital camera using a light microscope. The mean number of migrating cells was calculated from 5 randomly selected fields.

### RNA extraction and quantitative reverse transcription-polymerase chain reaction

Total RNA was isolated from tissues and cells using the TRIzol (Invitrogen) method according to the manufacturer’s protocols. The quality and quantity of RNA were examined using ND-1000 spectrophotometer (NanoDrop Technologies Inc.). A total of RNA was reversely transcribed into cDNA using a High-Capacity cDNA Reverse Transcription Kit (Applied Biosystems). The RT-PCR was performed using the SYBR Green qPCR Master Mix (Thermo Fisher Scientific) by the ABI PRISM 7300 real-time PCR system (Applied Biosystems). The endogenous controls of XIST was GAPDH, and the endogenous controls of miR-25-3p was U6. The thermal cycle was set as follows: 95°C for 1 minute and 40 cycles at 95°C for 15 seconds, 58°C for 20 seconds and 72°C for 20 seconds. The final expression values were calculated by the 2^-△△Ct^ method for subsequent analysis.

### Western blot (WB) analysis

E-cadherin is an epithelial cell marker, mainly involved in cell adhesion and maintaining the structural integrity of tissues.^23^ During the EMT process, the expression of E-cadherin is usually downregulated, which is regarded as one of the important indicators of EMT occurrence.^24^ N-cadherin is a marker for nerve and mesenchymal cells, and its expression is usually upregulated during the EMT process.^24^ Vimentin is an intermediate filament protein, mainly present in mesenchymal cells. Its expression is usually upregulated during the EMT process.^23^ These marker changes have been widely verified in the EMT process of various tumor cells and are widely used in EMT research.^24^

HOF cells were infiltrated with 200 µL of RIPA lysate. The lysate was vortexed briefly and subsequently centrifuged at 12,000 rpm (12,800 g) for 10 minutes. The cell supernatant was collected and the total protein concentration was determined by BCA assay. Equal amounts of protein were run on 10% SDS-PAGE, and proteins were electro-transferred onto polyvinylidene difluoride (PVDF) membranes (Millipore). The membrane containing the target protein was blocked with 5% (w/v) bovine serum albumin (BSA) and then incubated with the corresponding primary antibody overnight at 4°C. After incubation with horseradish peroxidase (HRP) -labelled secondary antibody for 3 hours at room temperature. The relative expression of the target proteins was measured by ImageJ (Rawak Software, Inc.) and normalised to the relative expression of β-actin. The WB tests were independently repeated 3 times, and for each experiment, 3 duplicate wells were set up.

### Dual-luciferase reporter assay

The sequences of XIST containing miR-25-3p binding sites were subcloned into dual luciferase reporter plasmids (Promega, USA) to construct wilde type recombinant luciferase reporter plasmids XIST -Wt, and the mutation sequences of the binding sites were inserted into the plasmids to construct mutant luciferase reporter plasmids XIST-Mut. HOFs incubated as above described were inoculated into 24-well plates, and the above recombinant plasmids were mixed with miR-25-3p mimic, miR-25-3p inhibitor, mimic NC, or inhibitor NC, respectively, and transfected under Lipofectamine 3000. After 48 hours, cells were harvested using the lysate from a commercial kit and relative luciferase activity was measured.

### Statistical analysis

All the data in this study were statistically analysed using GraphPad Prism 9.0 software. All experiments were repeated 3 times. The independent sample t-test was used for comparison between the 2 groups. One-way ANOVA was used for comparison among multiple groups, and Tukey's method was used for multiple comparisons among groups. All quantitative data are described by SD, which reflects the dispersion of the data and intuitively shows the variability within the groups. *P* < .05 indicates that the difference is statistically significant.

## Results

### Effects of curcumin on HOF cell biological behaviours and the TGF-β1/Smad pathway

**Fig. 1:**
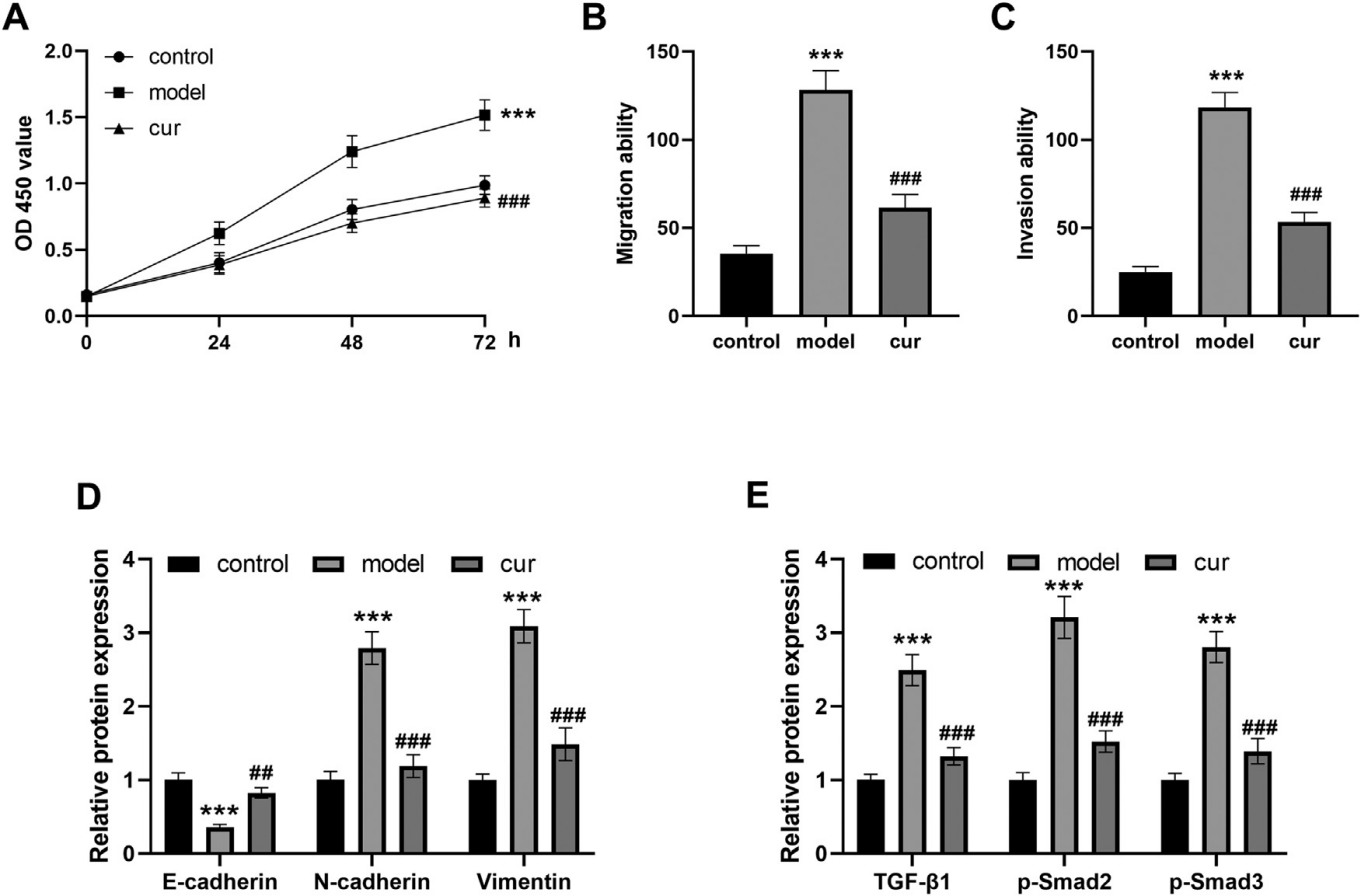
Curcumin can regulate the activity of HOF cells and alleviate the progression of OSF. The experiment was divided into 3 groups: the control group (control), the OSF model group induced by arecoline (model), and the curcumin intervention group after OSF model induction by arecoline (cur). (A) Cell proliferation was detected by CCK-8 assay. (B and C) Transwell assay was used to detect cell migration and invasion. (D and E) WB was used to detect the protein levels of EMT markers and TGF-β1/Smads pathway. *** *P* < .001 vs control; ## *P* < .01, ### *P* < .001 vs model. Data were presented as the mean ± SD (*n* = 3).

Results Figure 1A-C showed that the proliferation, migration, and invasion ability of the OSF cell model established by arecoline was significantly higher than that of the control group (all *P* < .001). Compared with the model group, the curcumin intervention group had significant reductions in cell viability (all *P* < .001).

At the same time, the expression of EMT marker E-cadherin in the model group was significantly decreased, and in the curcumin intervention group was significantly increased. The expressions of N-cadherin and Vimentin in the model group were significantly increased, while those in the curcumin group were significantly decreased (Fig 1D, all *P* < .01).

WB was used to detect the expression levels of TGF-β1, p-Smad2 and p-Smad3, the key molecules of TGF-β1/Smad pathway (Fig. 1E). The results showed that the expression of TGF-β1, p-Smad2 and p-Smad3 were significantly increased in the model group (*P* < .001), and decreased in the curcumin group (*P* < .001).

### Curcumin regulates the expression levels of XIST and miR-25-3p

**Fig. 2:**
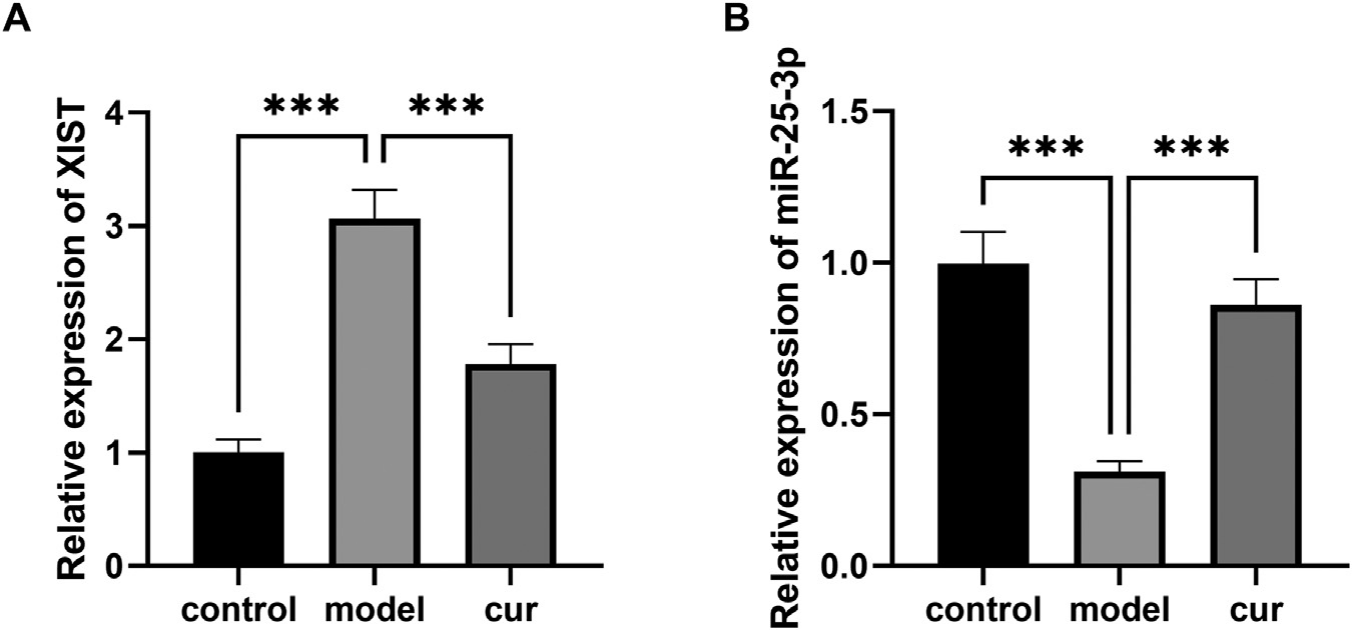
Curcumin down-regulated XIST and relieved the inhibition of miR-25-3p. The experiment was divided into 3 groups: the control group (control), the OSF model group induced by arecoline (model), and the curcumin intervention group after OSF model induction by arecoline (cur). (A) The expression of XIST was detected by PCR (*** *P* < .001). (B) The expression of miR-25-3p was detected by PCR (*** *P* < .001). Data were presented as the mean ± SD (*n* = 3).

qPCR results (Fig. 2) showed that arecoline up-regulated XIST and down-regulated miR-25-3p (*P* < .001). These effects were reversed by curcumin, which reduced XIST levels and increased miR-25-3p expression levels (*P* < .001).

### miR-25-3p was adsorbed by XIST sponge

**Fig. 3:**
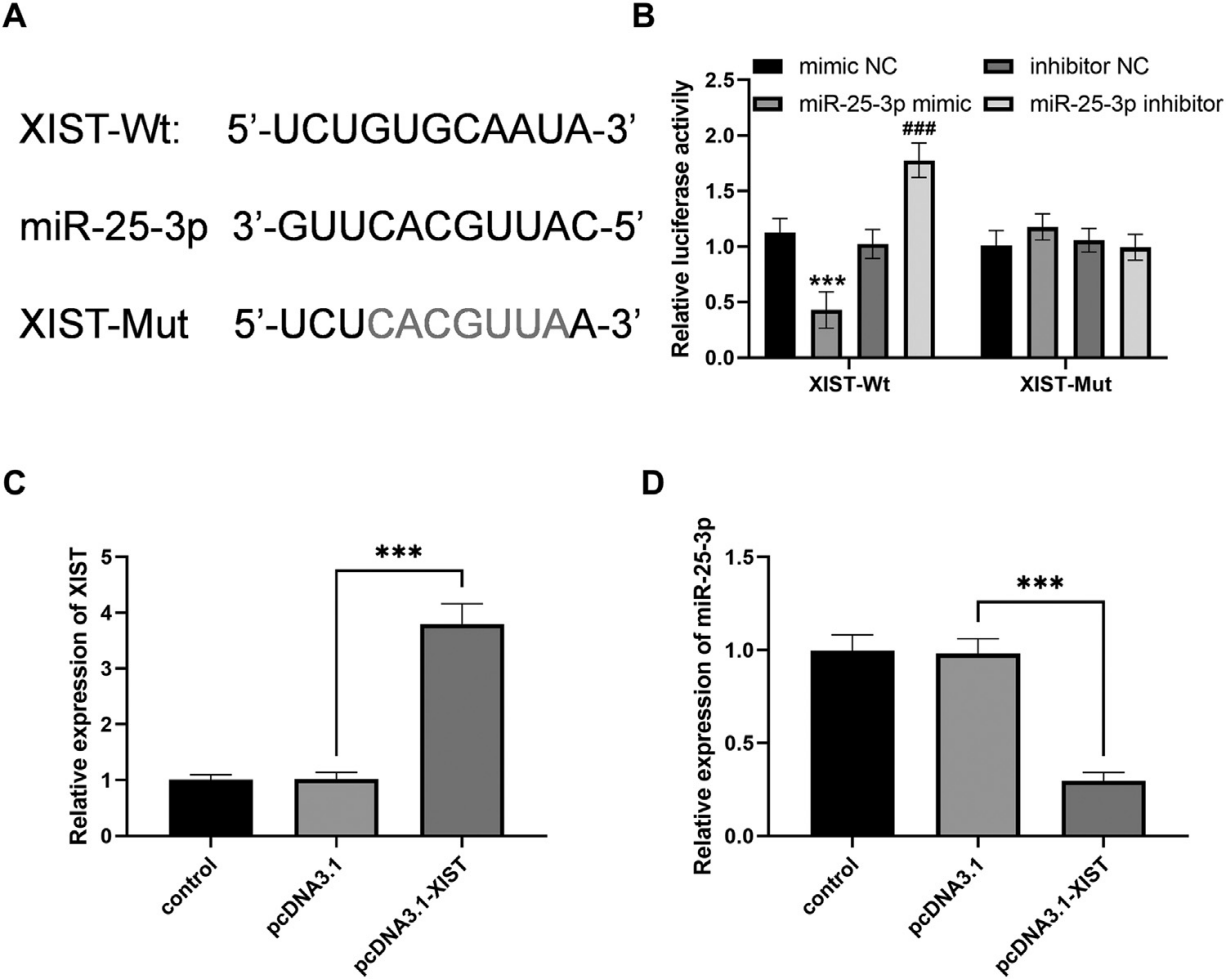
miR-25-3p is a Molecular Sponge for XIST. (A) The binding sites between XIST and miR-25- 3p. (B) Dual luciferase reporter assay validates target binding between miR-25-3p and XIST (*** *P* < .001 vs mimic NC; ### *P* < .001 vs inhibitor NC). (C) The expression of XIST was detected by PCR (*** *P* < .001). (D) The expression of miR-25-3p was detected by PCR (*** *P* < .001). Data were presented as the mean ± SD (*n* = 3).

We identified the binding site of XIST to miR-25–3p through a bioinformatics database (Fig. 3A). Dual-luciferase reporter gene assay was performed to assess the interaction between XIST and miR-25–3p. Dual luciferase reporter assay showed that miR-25–3p mimics significantly inhibited luciferase activity of XIST-Wt reporter gene, while miR-25–3p inhibitor led to the opposite result in HOFs (Fig. 3B, all *P* < .001). And the luciferase activity of XIST-Mut reporter gene was not changed (Fig. 3B, all *P* > .05).

The expression level of XIST was significantly increased after transfection with pcDNA3.1-XIST (Fig. 3C, *P* < .001). In addition, overexpression of XIST significantly reduced the expression level of miR-25-3p (Fig. 3D, *P* < .001).

### Curcumin regulates the activity of HOF cells through XIST/miR-25-3p

**Fig. 4:**
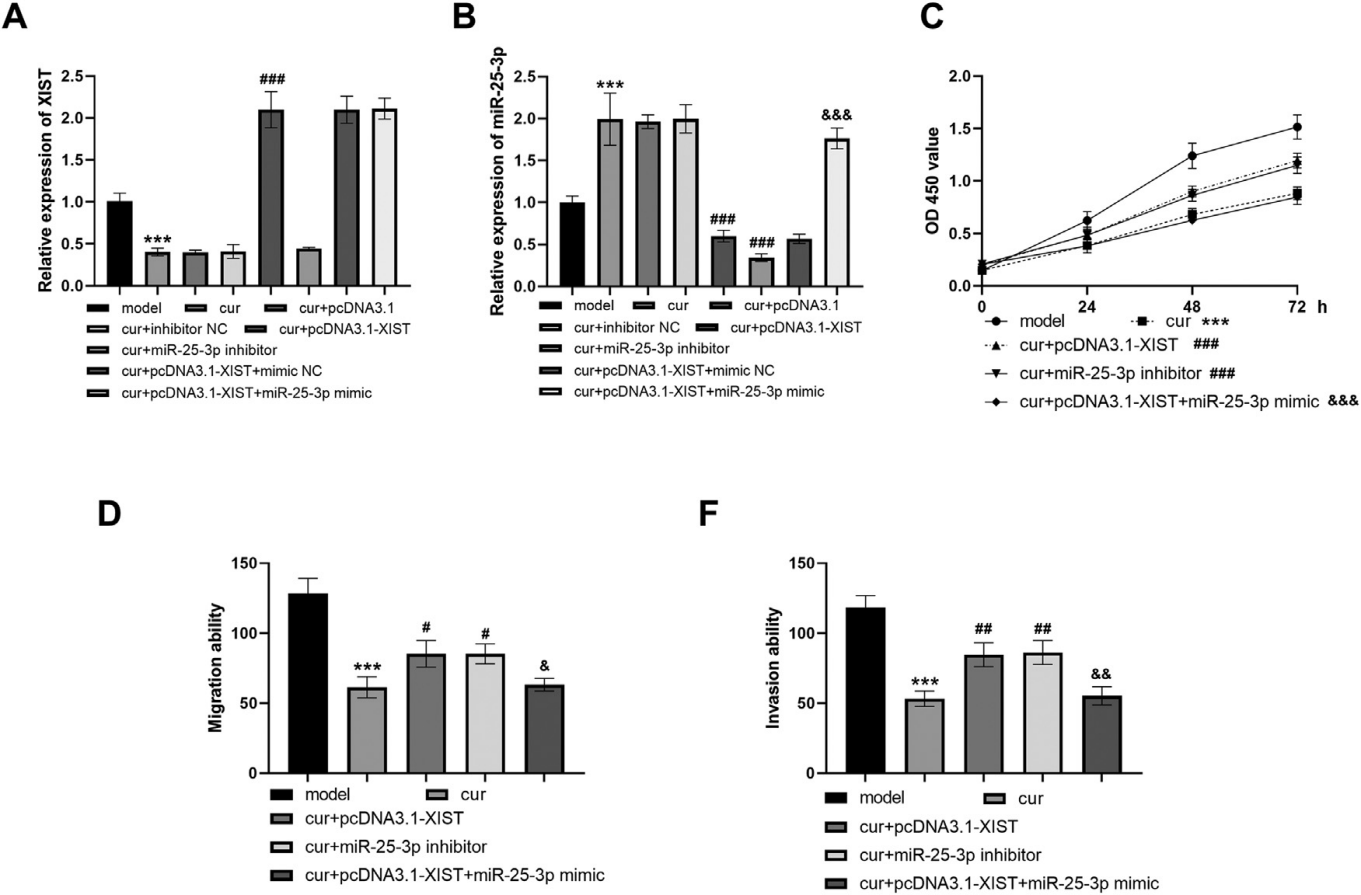
Curcumin inhibits the proliferation, migration and invasion of HOFs by regulating XIST/miR-25-3p. (A) The expression level of XIST after cell transfection treatment was detected by PCR. (B) The expression level of miR-25-3p. (C) Cell proliferation capacity. (D) Cell migration ability. (E) Cell invasion ability. *** *P* < .001 vs model; # *P* < .05, ## *P* < .01, ### *P* < .001 vs cur; & *P* < .05, && *P* < .01, &&& *P* < .001 vs cur+ pcDNA3.1-XIST. Data were presented as the mean ± SD (*n* = 3).

The results in Figure 4A and B showed that XIST expression was decreased and the expression of miR-25-3p was increased in HOF cells treated with curcumin (all *P* < .001). After transfection with pcDNA3.1-XIST, the expression of XIST was up-regulated and the expression of miR-25-3p was down-regulated (all *P* < .001). miR-25-3p inhibitor could reduce the expression level of miR-25-3p (*P* < .001). The negative control group (cur+pcDNA3.1 and cur+inhibitor NC) showed no significant differences in XIST expression and miR-25-3p expression compared to the cur group (*P* > .05). The cur+pcDNA3.1-XIST+mimic NC group and the cur+pcDNA3.1-XIST group also showed no significant differences in XIST and miR-25-3p expression (*P* > .05).

The results in Figure 4C-E showed that cell proliferation, migration and invasion were inhibited after curcumin treatment. Transfection of pcDNA3.1-XIST or miR-25-3p inhibitor increased the ability of cells to be inhibited by curcumin (Fig. 4C-E, all *P* <0.05). Co-transfection of pcDNA3.1-XIST and miR-25-3p mimic resulted in a decrease in cell viability compared with the pcDNA3.1-XIST group (Fig. 4C-E, all *P* < .05).

### Curcumin regulates EMT process through XIST/miR-25-3p

**Fig. 5:**
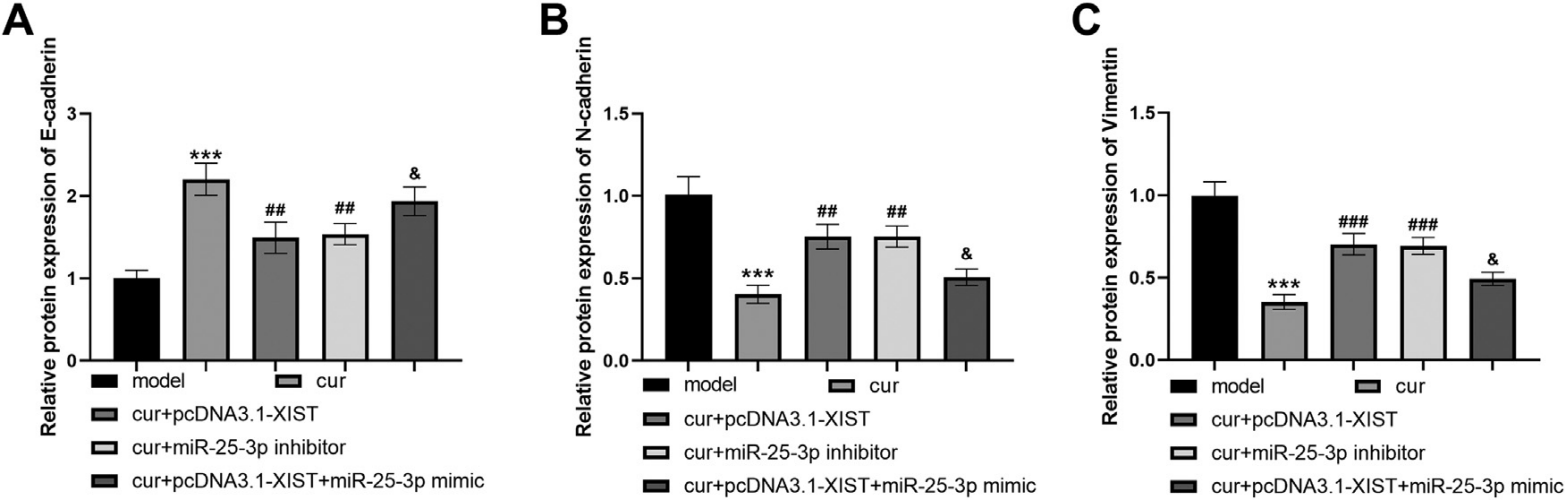
Curcumin regulates EMT process through XIST/miR-25-3p axis. The experiment was divided into 5 groups: the OSF model group induced by arecoline (model), the group intervened with curcumin after arecoline-induced OSF model (cur), the group transfected pcDNA3.1-XIST after curcumin intervention in the arecoline-induced OSF model (cur+ pcDNA3.1-XIST), the group transfected miR-25-3p inhibitor after curcumin intervention in the arecoline-induced OSF model (cur+ miR-25-3p inhibitor), and the group transfected both pcDNA3.1-XIST and miR-25-3p mimic after curcumin intervention in the arecoline-induced OSF model (cur+ pcDNA3.1-XIST+ miR-25-3p mimic). (A) Epithelial marker E-cadherin protein expression. (B) Mesenchymal marker N-cadherin protein expression. (C) Mesenchymal marker Vimentin protein expression. *** *P* < .001 vs model; ## *P* < .01, ### *P* < .001 vs cur; & *P* < .05 vs cur+ pcDNA3.1-XIST.

The expression of EMT markers was detected by WB. Compared with the arecoline induced model group, the curcumin group had increased expression of E-cadherin and decreased expression of N-cadherin and Vimentin (Fig. 5A-C, all *P* < .001). Overexpression of XIST and miR-25-3p inhibitor altered the expression levels of E-cadherin, N-cadherin and Vimentin after curcumin treatment (Fig. 5A-C, all *P* < .01). However, miR-25-3p mimic rescued the decreased expression of E-cadherin and increased expression of N-cadherin and Vimentin caused by XIST overexpression (Fig. 5A-C, all *P* < .05).

### Curcumin regulates TGF-β1/Smads signalling pathway through XIST/miR-25-3p

**Fig. 6:**
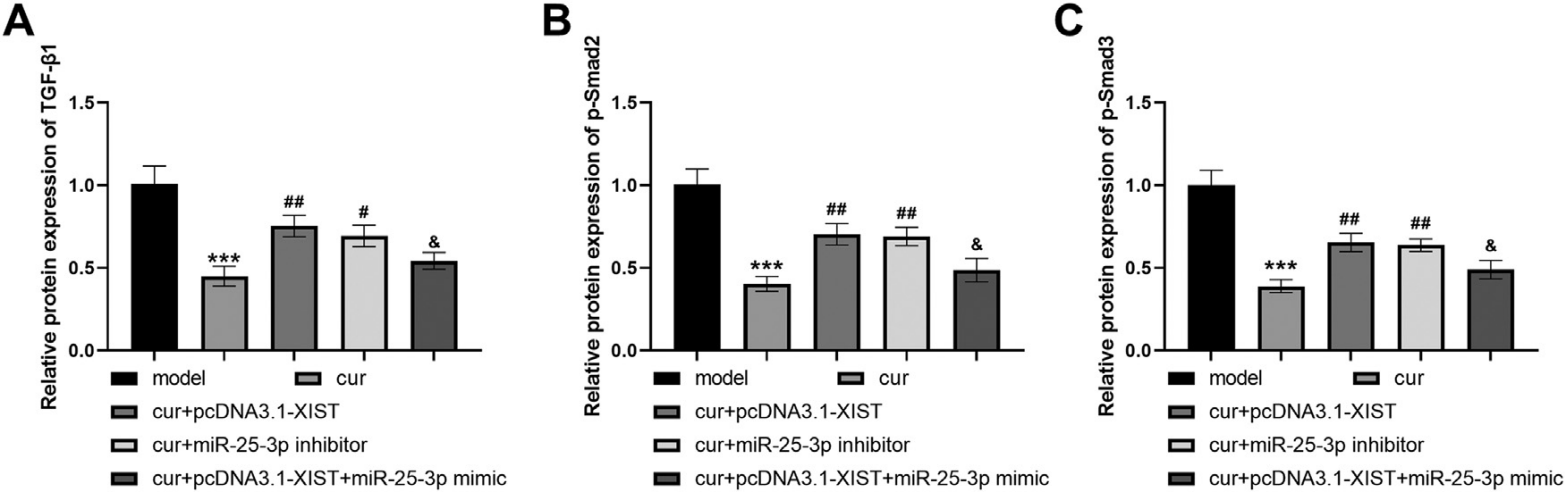
Curcumin inhibits TGF-β1/Smad signalling through the XIST/miR-25-3p axis. The experiment was divided into 5 groups: the OSF model group induced by arecoline (model), the group intervened with curcumin after arecoline-induced OSF model (cur), the group transfected pcDNA3.1-XIST after curcumin intervention in the arecoline-induced OSF model (cur+ pcDNA3.1-XIST), the group transfected miR-25-3p inhibitor after curcumin intervention in the arecoline-induced OSF model (cur+ miR-25-3p inhibitor), and the group transfected both pcDNA3.1-XIST and miR-25-3p mimic after curcumin intervention in the arecoline-induced OSF model (cur+ pcDNA3.1-XIST+ miR-25-3p mimic). (A) TGF-β1 protein expression levels. (B) p-Smad2 levels. (C) p-Smad3 levels. *** *P* < .001 vs model; # *P* < .05, ## *P* < .01 vs cur; & *P* < 0.05 vs cur+ pcDNA3.1-XIST.

The expressions of TGF-β1, p-Smad2 and p-Smad3 were decreased in the curcumin group, while XIST overexpression and miR-25-3p inhibitor increased the expression of key proteins in the TGF-β1/Smads signalling pathway inhibited by curcumin (Fig. 6A-C, all *P* < .05). Similarly, miR-25-3p mimics rescued the negative effects of XIST overexpression (Fig. 6A-C, all *P* < .05).

## Discussion

OSF is a chronic disease that seriously endangers oral health and has a tendency of cancerisation. The occurrence and development of OSF is a complex pathological process, in which EMT plays a key role.^25^ TGF-β1 has been shown to be an important EMT inducer in a variety of tissue and disease contexts.^26^^,^^27^ It can promote the progression of OSF by activating the TGF-β1/Smads pathway.^28^^,^^29^ This study aims to investigate the mechanism by which curcumin inhibits EMT process and relives OSF through XIST/miR-25-3p-mediated inactivation of TGF-β1/Smads pathway.

Multiple studies have confirmed that 30-50 μmol/L curcumin can significantly inhibit fibroblast activation and extracellular matrix deposition.^30^ This concentration can be effectively achieved in clinical local intervention. In the research on oral-related cells, 40 μmol/L curcumin showed no significant toxicity to normal oral mucosal cells.^31^ Further support the applicability of this concentration in the research of oral diseases. The local application of curcumin preparations shows significant anti-inflammatory effects and high safety in the treatment of oral mucosal inflammation and ulcers, etc.^32^^,^^33^ This provides a basis for the clinical translatability feasibility of the concentration used in this study. It is a reasonable concentration that takes into account the efficacy, toxicity, and correlation with the mechanism.

In the pathological development of OSF, the abnormal proliferation of HOF cells is an important factor leading to excessive deposition of fibrous tissue.^34^ TGF-β1/Smads signalling pathway is the core pathway to regulate EMT, and its activation can promote the transformation of epithelial cells to mesenchymal cells and accelerate the synthesis and accumulation of extracellular matrix.^35^^,^^36^ The results of the present study clearly showed that curcumin could effectively inhibit HOF cell viability and EMT process, while blocking the activation of TGF-β1/Smads signalling pathway. The inhibitory effect of curcumin on the above processes echoes previous findings in other fibrotic diseases. For example, in liver fibrosis studies, curcumin can reduce hepatic stellate cell activation and collagen deposition by inhibiting the TGF-β1/Smads signalling pathway.^37^ This study further confirmed that curcumin could play a direct intervention role in the treatment of OSF from multiple levels, such as cell proliferation, EMT process and key signalling pathways, and effectively curb the pathological progression of OSF, providing a solid cytological basis for curcumin as a potential therapeutic drug.

All the recombinant vectors used in this study (including XIST overexpression plasmid, miR-25-3p mimics and inhibitors) were verified for their intervention efficiency through qRT-PCR. Efficiency of XIST overexpression: The mRNA level of XIST in HOF cells was significantly higher than that in the pcDNA3.1 control group (about 3-fold, *P* < .01). Efficiency of miR-25-3p mimics: The expression level of miR-25-3p was significantly higher than that in the negative control group (about 2.6-fold, *P* < .01). Efficiency of miR-25-3p inhibitors: The expression level of miR-25-3p was significantly lower than that in the negative control group (about 0.5-fold, *P* < .01). The results showed that curcumin could precisely regulate the expression levels of XIST and miR-25-3p, and XIST could bind to miR-25-3p and regulate its expression through sponge adsorption, which revealed a new molecular mechanism of curcumin. XIST is involved in the regulatory network of gene expression in a variety of diseases, especially in fibrotic diseases.^38^ After curcumin treatment, the expression of XIST was significantly decreased, and the expression of miR-25-3p was significantly increased, indicating that curcumin could reshape the downstream gene regulatory network by regulating the expression of these 2 non-coding RNAs and their interaction.

Previous studies have shown that miR-25-3p regulates cell proliferation, migration, and invasion by targeting PTEN, BTG2 or FBXW7.^39^, ^40^, ^41^ When XIST expression is inhibited by curcumin, the expression level of miR-25-3p is increased, which in turn may target and regulate genes related to HOF cell activity. In this study, curcumin may inhibit the abnormal proliferation of HOF cells, reduce cell activity, and reduce the formation of fibrous tissue through XIST/miR-25-3p signalling axis. This finding further defines the molecular mechanism by which curcumin regulates cell activity in the treatment of OSF and also provides a theoretical basis for the development of new therapeutic strategies targeting the XIST/miR-25-3p signalling axis.

TGF-β1/Smads signalling pathway is a classical pathway that regulates EMT. TGF-β1 activates Smad proteins and promotes the transformation of epithelial cells to mesenchymal cells, which plays a key role in the occurrence and development of OSF.^42^ Curcumin could regulate the XIST/miR-25-3p signalling axis, increase the expression level of miR-25-3p, effectively inhibit the expression of TGF-β1, and inactivate the TGF-β1/Smads signalling pathway, thereby inhibiting the occurrence and development of EMT process from the root. Based on the multi-target regulatory characteristics of curcumin mentioned in the research by Diomede F et al., this study elucidates the common patterns of its mechanism in conjunction with the intervention of curcumin on pathways such as NF-κB and STAT3, emphasising the rationality of its action through non-single targets.^43^ Idrees M's article focuses on the application basis of curcumin in oral diseases, linking the HOF cell experiment results of this study with the clinical potential of local anti-inflammatory and anti-fibrotic effects in the oral cavity, strengthening the logical chain from basic research to clinical application.^44^ This suggested that curcumin is not a single target in the treatment of OSF, but through the complex regulatory network of XIST/miR-25-3p/TGF-β1/Smads, multi-link and multi-target cooperation to achieve effective intervention on the pathological process of OSF. The clarification of this mechanism provides a new theoretical basis and potential therapeutic target for the treatment of OSF, which is of great significance to promote the innovation and development of OSF treatment strategies.

The translational value and clinical application challenges of curcumin in OSF treatment. OSF is a chronic fibrotic disease closely related to exposure to arecoline, and currently lacks specific therapeutic drugs. This study confirmed that curcumin can inhibit the EMT process and abnormal proliferation of fibroblasts by regulating the XIST/miR-25-3p/TGF-β1/Smads pathway. It directly targets the core pathological mechanism of OSF (fibrosis progression and cancer risk), providing a potential targeted treatment strategy. Curcumin, as a natural plant extract, has been used in traditional medicine for a long time, and multiple preclinical studies have shown that it has low toxicity. This lays a certain safety foundation for its transformation from basic research to clinical application. The treatment of OSF often involves various methods such as surgery and drugs (such as glucocorticoids), but the effect is limited and prone to recurrence. Curcumin can form a synergistic effect with existing treatment methods by regulating fibrosis-related signalling pathways, thereby improving the overall treatment effect. Curcumin has the characteristics of poor lipid solubility and rapid metabolism, resulting in extremely low oral bioavailability. This means that a higher dose is required to reach an effective therapeutic concentration in the body, which may increase the risk of adverse reactions. Currently, there is a lack of specific formulations of curcumin for local use in the oral cavity. The lesions of OSF are mainly located in the submucosa of the oral cavity, and the drug is difficult to accumulate locally when administered systemically; while common local preparations (such as mouthwashes) are easily diluted by saliva and difficult to maintain an effective concentration. Therefore, developing new delivery systems with sustained release and targeted characteristics (such as nanocarriers, bioadhesive preparations) is a key challenge for its clinical transformation. Currently, studies on the treatment of OSF with curcumin mostly remain at the cellular and animal levels and lack large-scale, randomised controlled clinical research data. The optimal dose, treatment course, long-term safety, and impact on cancer risk of curcumin in the human body have not been clarified, and these require further clinical verification to promote its clinical application.

In the present study, it was found that curcumin was able to significantly inhibit arecoline induced EMT in HOF cells, as indicated by up-regulation of the epithelial marker E-cadherin and down-regulation of the mesenchymal markers N-cadherin and Vimentin. Further studies revealed that this effect of curcumin was achieved by regulating XIST/miR-25-3p-mediated TGF-β1/Smads pathway. This study also had some limitations. Firstly, the singularity of the cell model. OSF is a complex pathological process involving interactions among various cell types such as epithelial cells, fibroblasts, and immune cells. This study focuses solely on a single-cell model of HOF cells and cannot fully simulate the cross-regulation among cells in the microenvironment within the body. Secondly, the insufficiency of microenvironment simulation. The fibrotic microenvironment of OSF tissues in the body not only contains cell components but also involves dynamic remodeling of the extracellular matrix (such as cross-linking of collagen fibers, increase in matrix hardness), hypoxia, and chronic inflammatory states. However, the in vitro culture system is unable to replicate these physiological and pathological features, which may result in differences between the effects observed of curcumin in vitro and the actual situation in vivo. In view of these limitations, future studies can further validate and expand the conclusions of this research through in vivo studies. The OSF model in rats/mice was established by injecting or applying arecoline alkaloid. Curcumin was administered orally or topically to observe its effects on the degree of oral mucosal fibrosis (such as collagen deposition area, tissue hardness), expression of EMT markers, and activity of the TGF-β1/Smads pathway. The intervention effect of curcumin in vivo was verified. Through immunohistochemistry, single-cell sequencing and other techniques, the regulatory effects of curcumin on various cell types (epithelial cells, fibroblasts, macrophages) in OSF tissues were analysed. The cell specificity and overall network mechanism of its action in the in vivo microenvironment were clarified.

In conclusion, curcumin may effectively inhibit the progression of EMT by regulating the expression of XIST/miR-25-3p and mediating the inactivation of the TGF-β1/Smads pathway, thereby potentially alleviating the progression of OSF. This study provides a new theoretical basis and potential therapeutic target for the application of curcumin in the treatment of OSF, and may open up new ideas for the treatment of OSF.

## Author contributions

QM and YL carried out the research design and conception; JL and YZ analysed and interpreted the data regarding; QM and JL performed the examination of sample; YL contributed essential reagents or tools; QM and JQ authors wrote and revised the manuscript. All authors read and approved the final manuscript.

## Ethics approval and consent to participate

The experimental procedures were all in accordance with the guideline of the Ethics Committee of Changsha Stomatological Hospital and has approved by the Ethics Committee of Changsha Stomatological Hospital. This study complies with the Declaration of Helsinki.

A signed written informed consent was obtained from each patient.

## Consent for publication

Not applicable.

## Availability of data and materials

The data used and analysed can be obtained from the corresponding author under a reasonable request.

## Funding

Natural Science Foundation of Hunan Province: Study on Curcumin mediating Epithelial-endothelial-Mesenchymal trans differentiation against Oral Submucosal Fibrosis based on the TGF-β/Smads signalling Pathway (ID: 2025JJ80534)

Health Commission of Changsha City: Effects of paeoniflorin on rat oral submucous fibrosis and TGF-β-induced collagen secretion in fibroblasts (ID: KJ-B2023082)

## Conflict of interest

None disclosed.
